# Ubiquitination of *Listeria* Virulence Factor InlC Contributes to the Host Response to Infection

**DOI:** 10.1128/mBio.02778-19

**Published:** 2019-12-17

**Authors:** Edith Gouin, Damien Balestrino, Orhan Rasid, Marie-Anne Nahori, Véronique Villiers, Francis Impens, Stevenn Volant, Thomas Vogl, Yves Jacob, Olivier Dussurget, Pascale Cossart

**Affiliations:** a Institut Pasteur, Unité des Interactions Bactéries-Cellules, Paris, France; b Inserm, U604, Paris, France; c INRA, USC2020, Paris, France; d Institut Pasteur, Groupe à 5 ans Chromatine et Infection, Paris, France; e Institut Pasteur, Hub Bioinformatique et Biostatistique, Paris, France; f CNRS, USR3756, Paris, France; g Institute of Immunology, University of Münster, Münster, Germany; h Institut Pasteur, Unité de Génétique Moléculaire des Virus à ARN, Paris, France; i CNRS, UMR3569, Paris, France; j Université Paris Diderot, Sorbonne Paris Cité, Paris, France; University of Illinois at Chicago

**Keywords:** *Listeria monocytogenes*, ubiquitination, pathogenesis, alarmin, inflammation

## Abstract

Listeria monocytogenes is a pathogenic bacterium causing potentially fatal foodborne infections in humans and animals. While the mechanisms used by *Listeria* to manipulate its host have been thoroughly characterized, how the host controls bacterial virulence factors remains to be extensively deciphered. Here, we found that the secreted *Listeria* virulence protein InlC is monoubiquitinated by the host cell machinery on K224, restricting infection. We show that the ubiquitinated form of InlC interacts with the intracellular alarmin S100A9, resulting in its stabilization and in increased reactive oxygen species production by neutrophils in infected mice. Collectively, our results suggest that posttranslational modification of InlC exacerbates the host response upon *Listeria* infection.

## Abstract (precis)

The pathogenic potential of Listeria monocytogenes relies on the production of an arsenal of virulence determinants that have been extensively characterized, including surface and secreted proteins of the internalin family. We have previously shown that the *Listeria* secreted internalin InlC interacts with IκB kinase α to interfere with the host immune response (E. Gouin, M. Adib-Conquy, D. Balestrino, M.-A. Nahori, et al., Proc Natl Acad Sci USA, 107:17333–17338, 2010, https://doi.org/10.1073/pnas.1007765107). In the present work, we report that InlC is monoubiquitinated on K^224^ upon infection of cells and provide evidence that ubiquitinated InlC interacts with and stabilizes the alarmin S100A9, which is a critical regulator of the immune response and inflammatory processes. Additionally, we show that ubiquitination of InlC causes an increase in reactive oxygen species production by neutrophils in mice and restricts *Listeria* infection. These findings are the first to identify a posttranscriptional modification of an internalin contributing to host defense.

## INTRODUCTION

Listeria monocytogenes is a Gram-positive bacterium widespread in the environment and the etiological agent of listeriosis, a life-threatening foodborne disease. It is responsible for gastroenteritis in healthy individuals, meningitis, septicemia in immunocompromised individuals, miscarriages in pregnant women, and perinatal infections (1). Following ingestion of contaminated food, L. monocytogenes is able to cross the intestinal barrier, to reach the liver and spleen through the lymph and the bloodstream, and to disseminate to the brain and placenta after crossing the blood-brain barrier and the maternofetal barrier, respectively (2). At the cell level, this bacterium has the ability to invade, survive, and replicate intracellularly within professional phagocytes and a number of nonphagocytic cells, to spread from cell to cell, escaping host immunity. Upon infection of the host, L. monocytogenes employs a series of virulence factors that control host molecules and hijack cellular processes (3). The main invasion proteins, InlA and InlB, promote bacterial entry into nonphagocytic cells by interacting with the surface receptors E-cadherin and c-Met, respectively (4–7). The secreted listeriolysin O (LLO) and phospholipases (PlcA and PlcB) disrupt the primary and secondary vacuoles and allow the escape of the bacterium into the cytoplasm (8–10). ActA mimics the host actin polymerization machinery to promote motility, allowing intercellular spread and protection from autophagy (11, 12, 13). All the virulence factors cited above are positively controlled by the transcriptional activator PrfA (6, 13, 14). Inactivation of PrfA or that of the major virulence factors, e.g., LLO or ActA, leads to severe attenuation of *Listeria* virulence in animal models of infection (11, 13, 15). Other factors have a more moderate role in virulence, yet they are important for specific cellular processes, tissue types, or stages of the infectious process. This is the case for InlC, whose inactivation leads to a 0.5- to 1.5-log increase in the 50% lethal dose (LD_50_) after intravenous inoculation in mice (16, 17). InlC is a secreted protein of the *Listeria* internalin family whose expression is positively regulated by PrfA (18, 19). It is highly expressed by *Listeria* after its internalization inside host cells (20). InlC has several functions in host cells. We previously reported that it interacts with IKK-α, impairs the phosphorylation of IκB-α and delays the degradation of phospho-IκB-α, thereby impairing NF-κB translocation to the nucleus and subsequently dampening the host innate response, in particular genes encoding proinflammatory cytokines (16). Interestingly, InlC has also been shown to bind the scaffolding protein Tuba in polarized human cells (21). Tuba and its effectors N-WASP and CDC42 form a complex that generates tension at apical junctions of polarized cells. InlC inhibits the interaction between N-WASP and Tuba and thereby promotes relaxation of cortical actin tension, protrusion formation, and bacterial cell-to-cell spread.

S100A9, also named myeloid-related protein 14 (MRP14) or calgranulin B, is a member of the S100 family of calcium-binding proteins and forms a heterodimeric complex with S100A8, also known as MRP8 or calgranulin A, which is involved in inflammation (22, 23). This heterodimeric complex (calprotectin) is constitutively and highly expressed in neutrophils. In monocytes, macrophages, and endothelial cells, S100A8/S100A9 expression is induced by bacterial products such as lipopolysaccharide, proinflammatory cytokines such as tumor necrosis factor alpha (TNF-α), or anti-inflammatory cytokines such as interleukin-10 (IL-10) (24). These antimicrobial proteins act as danger signals in vertebrates and are called damage-associated molecular patterns (DAMPs) or alarmins. They indirectly prevent bacterial adhesion to the mucosal epithelium and inhibit bacterial growth through nutrient metal chelation (25). Clinical data show clear correlations between the concentration of these proteins in the serum or synovial fluid of patients with chronic inflammatory diseases and pathogenesis (26). In addition to their antimicrobial properties, these proteins have been shown to be involved in neutrophil activation and migration to inflammatory sites (23, 27).

In this study, we found that InlC is monoubiquitinated by the host cell machinery on K^224^, restricting infection. This ubiquitinated form of InlC interacts with and stabilizes the intracellular alarmin S100A9, resulting in increased reactive oxygen species (ROS) production by neutrophils in infected mice. Together, our data highlight that posttranslational modification of InlC contributes to the host response upon *Listeria* infection.

## RESULTS

### The L. monocytogenes InlC protein is ubiquitinated in infected epithelial cells.

**FIG 1:**
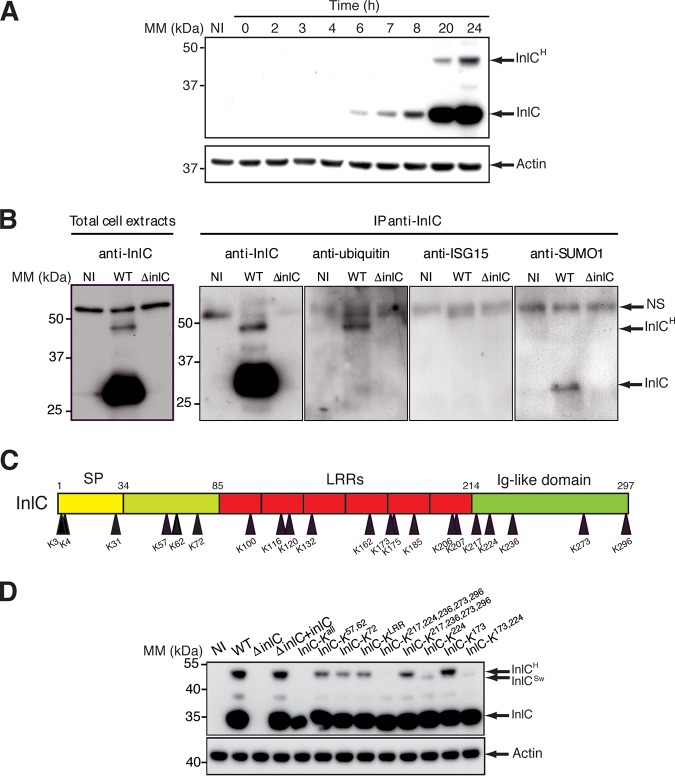
InlC is modified. (A) Time course analysis of InlC expression in cells infected with L. monocytogenes EGD. Total HeLa cell extracts at different time points after infection were analyzed by Western blotting using anti-InlC and antiactin antibodies. Actin is used as a loading control. The arrows highlight the main InlC forms, InlC and InlC^H^. MM, molecular mass. (B) InlC is ubiquitinated. Total cell lysates of HeLa cells, noninfected (NI) or infected with the wild-type (WT) or the *inlC* mutant (Δ*inlC*) strains, were immunoprecipitated (IP) with anti-InlC antibodies, followed by immunoblotting with antiubiquitin (mouse FK2, catalog number BML-PW8810; Enzo Life), anti-SUMO1 (catalog number 4930S; Cell Signaling Technology), or anti-ISG15 (mouse F-9, catalog number sc166755; Santa Cruz) antibodies. Note that the anti-SUMO1 antibodies recognized nonspecifically (NS) the InlC protein. (C) Schematic representation of InlC indicating the locations of lysine residues within the whole protein. SP, signal peptide; LRRs, leucine-rich repeats. (D) Identification of the InlC ubiquitination sites. Shown is the effect of mutations of InlC lysine residues on its expression in cells. Total cell lysates of HeLa cells, noninfected or infected for 24 h with the mutants, were immunoblotted with anti-InlC antibodies. Numbers indicate the positions of lysine residues according to the diagram shown in panel C. The data shown are representative of independent data from at least three experiments.

We reexamined the expression of InlC by L. monocytogenes during infection of HeLa cells. As we previously showed (16), InlC is gradually secreted during infection (Fig. 1A). In addition to the secreted InlC mature form (30 kDa, without a signal peptide), we observed, using immunoblot experiments with anti-InlC antibodies, other higher-molecular-weight species, including a major 47-kDa band (InlC^H^) (Fig. 1A). The difference in molecular weights between these proteins prompted us to examine whether these forms could correspond to InlC posttranslationally modified by ubiquitin or ubiquitin-like proteins (UBLs), such as interferon-stimulated gene 15 (ISG15) and SUMO. HeLa cells were infected with the wild-type (WT) L. monocytogenes EGD strain or a Δ*inlC* mutant for 24 h and lysed, and total cell extracts were immunoprecipitated with anti-InlC antibodies. Immunoprecipitates were analyzed by immunoblot experiments using anti-InlC, antiubiquitin, anti-ISG15, and anti-SUMO1 antibodies. As shown in Fig. 1B, the major larger form of InlC, i.e., InlC^H^, was detected only by antiubiquitin antibodies in cells infected with the WT strain but not in cells infected with the Δ*inlC* mutant and not by anti-UBL antibodies, suggesting that this form corresponds to ubiquitinated InlC.

### InlC protein is ubiquitinated on lysine 224.

In order to map the InlC ubiquitination site, we generated *Listeria* strains in which each of the 21 lysine residues of InlC were replaced, individually or in combination, by arginine residues (Fig. 1C). Two additional *Listeria* strains were generated, one in which all lysines have been replaced by an arginine (InlC-K^all^) and the other in which each lysine of the six leucine-rich repeats (LRRs) has been replaced by an arginine (InlC-K^LRR^). The different strains were tested for InlC ubiquitination in epithelial cells infected for 24 h. We noted that each strain expressed InlC at levels similar to that of the wild-type strain in HeLa cells, except for the InlC-K^all^ strain, which produced slightly less InlC (Fig. 1D). As expected, the InlC^H^ ubiquitinated form could not be detected in the InlC-K^all^ strain. Strikingly, the level of the InlC^H^ form appeared significantly decreased in the InlC-K^224^, InlC-K^173,224^, and InlC-K^217,224,236,273,296^ strains. Interestingly, InlC^H^ of the InlC-K^224^ mutant was replaced by a slightly smaller modified form (InlC^Sw^), possibly due to a modification on another lysine residue, a phenomenon previously observed for other UBL targets (28). However, the lysine K^173^, implicated in the binding of InlC to Tuba (21), was not a ubiquitination site, as the corresponding mutated InlC is still ubiquitinated. Together, these results demonstrate that InlC is ubiquitinated during infection of epithelial cells, and the major site of ubiquitination is the lysine residue K^224^.

### InlC is monoubiquitinated upon infection.

**FIG 2:**
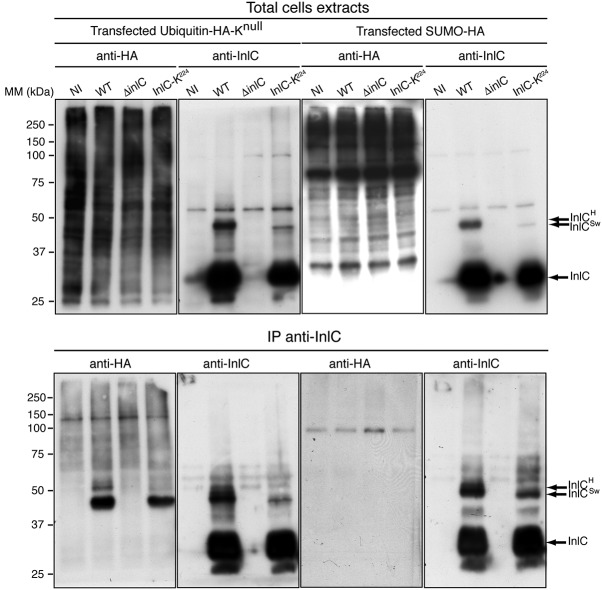
InlC is monoubiquitinated in epithelial cells upon infection. HeLa cells were transfected with K^null^-HA-ubiquitin or WT HA-SUMO1 and then were noninfected or infected with L. monocytogenes EGD WT, Δ*inlC*, or InlC-K^224^ for 24 h. Cell lysates were subjected to immunoprecipitation with anti-InlC antibodies, and immunocomplexes were analyzed by immunoblotting using anti-HA and anti-InlC antibodies. Input fractions are shown as controls. The data shown are representative of independent data from two experiments.

In order to determine whether InlC is mono- or polyubiquitinated, HeLa cells were transiently transfected with plasmids expressing hemagglutinin (HA)-tagged lysineless ubiquitin (K^null^), which lacks lysine residues and does not allow the formation of ubiquitin chains (29), or HA-tagged SUMO1 as a control (30). Cells were then infected with *Listeria* encoding native InlC or InlC-K^224^. After 24 h of infection, InlC was immunoprecipitated with anti-InlC antibodies. Immunoprecipitates were separated by SDS-PAGE, and the presence of ubiquitinated InlC was probed using anti-HA antibodies. The InlC^H^ form was detected in cells transfected with K^null^ ubiquitin and infected with *Listeria* encoding native InlC, suggesting that InlC^H^ corresponds to a monoubiquitinated InlC (Fig. 2). The InlC^Sw^ form was also detected in cells transfected with K^null^ ubiquitin and infected with bacteria expressing InlC-K^224^, reinforcing the hypothesis of a compensatory ubiquitination of another lysine residue upon K-to-R substitution of lysine 224. For cells transfected with HA-tagged SUMO1 and infected with *Listeria* encoding native InlC or InlC-K^224^, we did not observe, by immunoprecipitation, a SUMOylated form of InlC. Together, our results indicate that InlC can be ubiquitinated and that InlC^H^ corresponds to a monoubiquitination on lysine K^224^.

### InlC ubiquitination contributes to the host response to *Listeria* infection.

**FIG 3:**
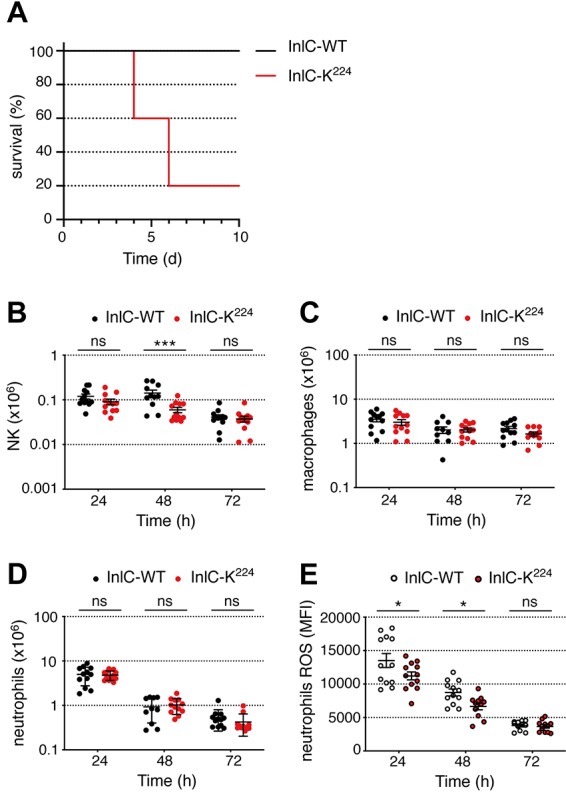
InlC ubiquitination restricts infection and modulates ROS production by neutrophils in mice. (A) BALB/c mice were infected by intravenous injection of 10^4^ bacteria of the L. monocytogenes EGD WT or InlC-K^224^ strains. Survival was determined daily for 10 days. Comparison of WT and InlC-K^224^ data was performed using the Mantel-Cox test. Survival curves were significantly different (*P ≤ *0.05). (B to E) C57BL/6 mice were infected by intraperitoneal injection of 10^7^ bacteria of the L. monocytogenes EGD WT or InlC-K^224^ strains. Data from two experiments were pooled. The numbers of NK cells (B), macrophages (C), and neutrophils (D) and the level of ROS produced by neutrophils (E) in peritoneal lavage fluids were assessed by flow cytometry at 24, 48, and 72 h postinfection. Comparison of WT and InlC-K^224^ data was performed using two-way analysis of variance and a Holm-Sidak *post hoc* test (α = 0.05). Mean values with a *P* value of ≤0.05 were statistically different (ns, nonsignificant; *, *P *≤ 0.05; ***, *P *≤ 0.001). The data shown are representative of independent data from two to five experiments. MFI, mean fluorescence intensity.

To evaluate if InlC ubiquitination plays a role in the infectious process *in vivo*, we compared the LD_50_s of L. monocytogenes EGD strains expressing WT InlC (InlC-WT) and those expressing InlC-K^224^ in female BALB/c mice infected by the intravenous (i.v.) route. Lysine 224 substitution led to an LD_50_ of 3 × 10^3^ CFU, 1 log_10_ unit lower than the LD_50_ of the wild-type strain, which was 3 × 10^4^ CFU. The survival of mice infected with 10^4^ L. monocytogenes bacteria expressing InlC-K^224^ was strongly reduced (Fig. 3A). In contrast, the whole group of mice infected with the wild-type strain survived and fully recovered. Thus, ubiquitination of InlC contributes to host protection against *Listeria*.

To investigate further the role of InlC ubiquitination in systemic infection, we infected female C57BL/6 mice intraperitoneally with 1 × 10^7^ bacteria of wild-type *Listeria* or the *Listeria* strain expressing InlC-K^224^. The number of bacteria, the number of immune cells, and ROS production by neutrophils in the peritoneal lavage fluid of infected animals were determined at 24, 48, and 72 h postinfection. Mutation of K^224^ in InlC did not lead to any significant change in the bacterial counts recovered from the lavage fluid at these time points (data not shown). We next measured the number of immune cells by flow cytometry in the same lavage fluids. At 24 h, infection of mice with *Listeria* expressing native InlC or InlC-K^224^ led to a similar recruitment of NK cells, macrophages, and neutrophils (Fig. 3B to D). At later time points, there was a decrease in the number of immune cells in the peritoneal lavage fluid of infected mice, which was marked for neutrophils and more moderate for macrophages and NK cells. As seen at 24 h, the K^224^ mutation did not affect significantly the number of phagocytic cells at 48 and 72 h postinfection (Fig. 3B to D). In contrast, the strain producing InlC-K^224^ triggered weaker ROS production by neutrophils than the WT strain (Fig. 3E). Together, these results demonstrate that InlC ubiquitination modulates the activation of neutrophils upon *Listeria* infection, without affecting the cell number significantly.

### InlC interacts with S100A9.

**FIG 4:**
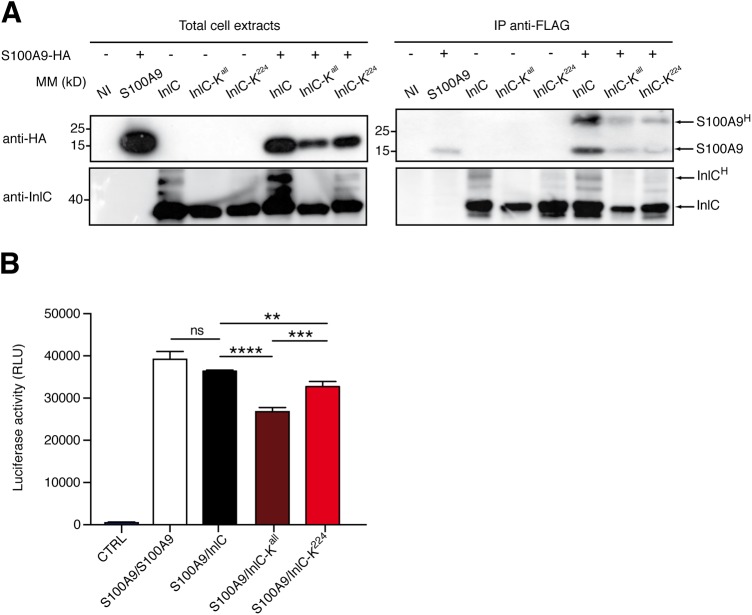
InlC interacts with S100A9. (A) Interaction of S100A9 with InlC in transfected HeLa cells. Whole-cell lysates from HeLa cells, nontransfected or transfected with the indicated plasmids, were immunoprecipitated with anti-FLAG beads, followed by immunoblotting with anti-InlC and anti-HA antibodies. S100A9^H^ corresponds to the dimeric form of the protein. The data shown are representative of independent data from three experiments. (B) Luciferase protein complementation assay. HEK-293T cells were transfected with 100 ng of plasmids expressing each part of the nanoluciferase alone (control [CTRL]) or S100A9 or InlC bearing NanoLuc moiety 1 or 2 at their N or C terminus. At 24 h posttransfection, the medium of the cells was discarded, and cells were incubated with 50 μl of Nano-Glo reagent. Luciferase enzymatic activity was measured using a Berthold Centro XS LB960 luminometer and counting luminescence for 2 s. The values represent the means from quadruplicate experiments. Statistical analysis was performed using a *t* test. Mean values with *P* values of ≤0.05 were statistically different (ns, nonsignificant; **, *P *≤ 0.01; ***, *P *≤ 0.001; ****, *P *≤ 0.0001). RLU, relative light units.

The two known roles of InlC, i.e., inhibition of NF-κB nuclear translocation (16) and promotion of cell-to-cell spread (21), were not affected by ubiquitination (data not shown). We thus assessed further the role of InlC modification during infection by searching putative partners that would interact with native or nonubiquitinatable InlC. Extracts from HeLa cells infected for 24 h with *Listeria* expressing native InlC, InlC-K^all^, or InlC-K^224^ were immunoprecipitated with anti-InlC antibodies, and the immunoprecipitates were analyzed by liquid chromatography-tandem mass spectrometry (LC-MS/MS), leading to the identification of the alarmin S100A9 as a putative interactor of InlC (see Table S1 in the supplemental material). To confirm this interaction, we transfected HeLa cells with vectors expressing humanized FLAG-tagged InlC, InlC-K^all^, or InlC-K^224^ and HA-tagged S100A9. Cell lysates were then immunoprecipitated with anti-FLAG magnetic beads, and immunoprecipitates were analyzed by immunoblotting with anti-InlC and anti-HA antibodies. As shown in Fig. 4A, S100A9 coimmunoprecipitated with InlC. Notably, larger amounts of S100A9 were detected following immunoprecipitation with native InlC than with InlC-K^all^ or InlC-K^224^.

10.1128/mBio.02778-19.3 TABLE S1 List of the proteins that were identified in the InlC pulldown experiments. The false discovery rate (FDR) for protein identification was set at 1% on the peptide-to-spectrum match (PSM), peptide, and protein levels. Columns from left to right contain the UniProt accession number of the MaxQuant majority protein, the gene name, the protein name, and an indication of whether the protein was identified in the InlC-K^224^, InlC-K^all^, or InlC-WT sample. Proteins are ranked alphabetically by protein name. Download Table S1, XLSX file, 0.1 MB. Copyright © 2019 Gouin et al. 2019 Gouin et al. This content is distributed under the terms of the Creative Commons Attribution 4.0 International license.

To further confirm the interaction between S100A9 and InlC, we designed a split-nanoluciferase (NanoLuc) protein complementation assay (NPCA). Plasmids expressing S100A9 or native InlC, InlC-K^all^, or InlC-K^224^ fused to two complementary NanoLuc moieties were cotransfected into cells, and luciferase activity was measured 24 h after transfection. Coexpression of S100A9 with InlC led to strong luciferase activity, similar to the intensity observed for S100A9/S100A9 homodimers, confirming the S100A9 interaction with InlC (Fig. 4B). Remarkably, the luciferase intensity was reduced when S100A9 was coexpressed with InlC-K^all^ or InlC-K^224^, in line with the immunoprecipitation data. This indicates that S100A9 interacts to a lesser extent with nonubiquitinatable and K^224^-substituted forms of InlC and, thus, that ubiquitination of InlC promotes its interaction with S100A9.

### Ubiquitination of InlC controls the level of S100A9 and the S100A9-dependent host response to infection.

**FIG 5:**
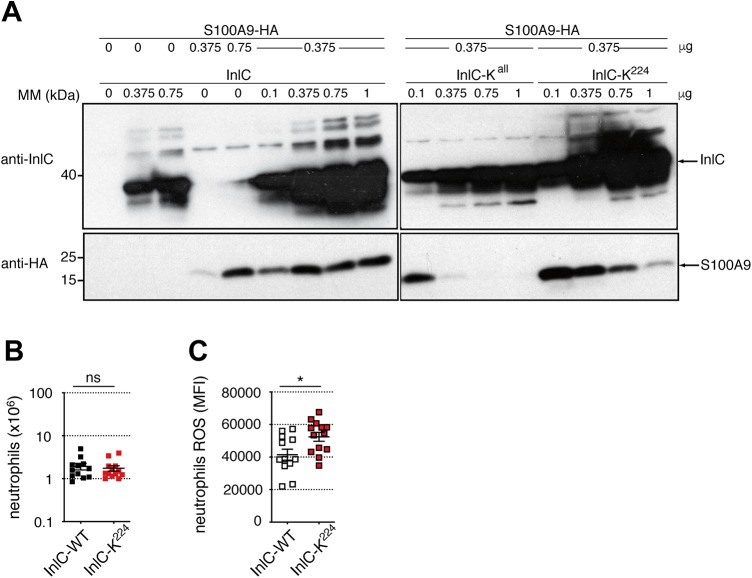
Ubiquitination of InlC controls the levels of S100A9 and ROS production by neutrophils in an S100A9-dependent manner. (A) HeLa cells were nontransfected or cotransfected with a plasmid encoding HA-tagged S100A9 and increasing amounts of a plasmid encoding either native InlC, InlC-K^all^, or InlC-K^224^. Whole-cell lysates were analyzed by immunoblotting with anti-InlC and anti-HA antibodies. The data shown are representative of independent data from three experiments. (B and C) S100A9^−/−^ mice were infected by intraperitoneal injection of 10^7^ bacteria of the L. monocytogenes EGD WT or InlC-K^224^ strains. The number of neutrophils (B) and the level of ROS produced by neutrophils (C) in peritoneal lavage fluids were assessed by flow cytometry at 24 h postinfection. Comparison of WT and InlC-K^224^ data was performed using a *t* test. Mean values with *P* values of ≤0.05 were statistically different (ns, nonsignificant; *, *P* ≤ 0.05). The data shown are representative of independent data from two experiments.

To investigate the functional significance of InlC ubiquitination, and to evaluate whether the interaction of ubiquitinated InlC with S100A9 could modify the level of S100A9, we cotransfected HeLa cells with a plasmid expressing HA-tagged S100A9 and a plasmid expressing either InlC, InlC-K^all^, or InlC-K^224^ at various concentrations. Cell extracts were analyzed by immunoblotting with anti-InlC and anti-HA antibodies. The levels of S100A9 increased when the InlC-WT plasmid was transfected (Fig. 5A). The levels of S100A9 remained stable upon further increased expression of InlC. In contrast, increasing InlC-K^all^ expression led to a massive decrease of S100A9, which dropped from detectable to undetectable levels when 0.1 to 0.75 μg of the InlC-K^all^ plasmid was transfected. Similarly, the expression of InlC-K^224^ led to the initial detection of S100A9, which progressively decreased when the production of InlC-K^224^ increased, suggesting that ubiquitination of InlC stabilizes S100A9 and prevents its disappearance. A similar trend was observed in HeLa cells transfected with a plasmid expressing HA-tagged S100A9, whose S100A9 levels were higher upon infection with *Listeria* expressing native InlC than upon infection with *Listeria* expressing InlC-K^all^ or InlC-K^224^ (Fig. S1).

10.1128/mBio.02778-19.1 FIG S1 Ubiquitination of InlC affects S100A9 levels in infected cells. HeLa cells were transfected with pcDNA3.1+/N-HA-S100A9 for 48 h and infected with L. monocytogenes EGD WT, InlC-K^all^, or InlC-K^224^ strains at an MOI of 60. After 24 h of stimulation with 50 ng/ml TNF-α, the levels of S100A9 in supernatants were measured by an ELISA. Comparisons of WT and InlC-K^all^ and of WT and InlC-K^224^ data were performed using a *t* test. Mean values with *P* values of ≤0.05 were statistically different (*P* values were 0.19 and 0.21, respectively). Experiments were repeated three times. Download FIG S1, TIF file, 0.3 MB. Copyright © 2019 Gouin et al. 2019 Gouin et al. This content is distributed under the terms of the Creative Commons Attribution 4.0 International license.

Given that host ubiquitination of InlC contributes to the host response to infection, we next sought to determine if S100A9 was involved in this response. We infected S100A9^−/−^ mice intraperitoneally with 1 × 10^7^ bacteria of wild-type *Listeria* or the *Listeria* strain expressing InlC-K^224^. The number of neutrophils and ROS production by neutrophils in the peritoneal lavage fluid of infected animals were determined at 24 h postinfection. Infection of mice with *Listeria* encoding InlC or InlC-K^224^ led to a similar recruitment of neutrophils (Fig. 5B). In contrast to what was observed in S100A9^+/+^ mice (Fig. 3E), in S100A9-deficient mice, the WT *Listeria* strain did not trigger a higher level of ROS production by neutrophils than the strain producing InlC-K^224^ (Fig. 5C). Together, these results demonstrate that InlC ubiquitination modulates the activation of neutrophils upon *Listeria* infection in an S100A9-dependent manner.

### Ubiquitinated InlC interacts with S100A9 partners and proteins involved in S100A9-dependent pathways.

**FIG 6:**
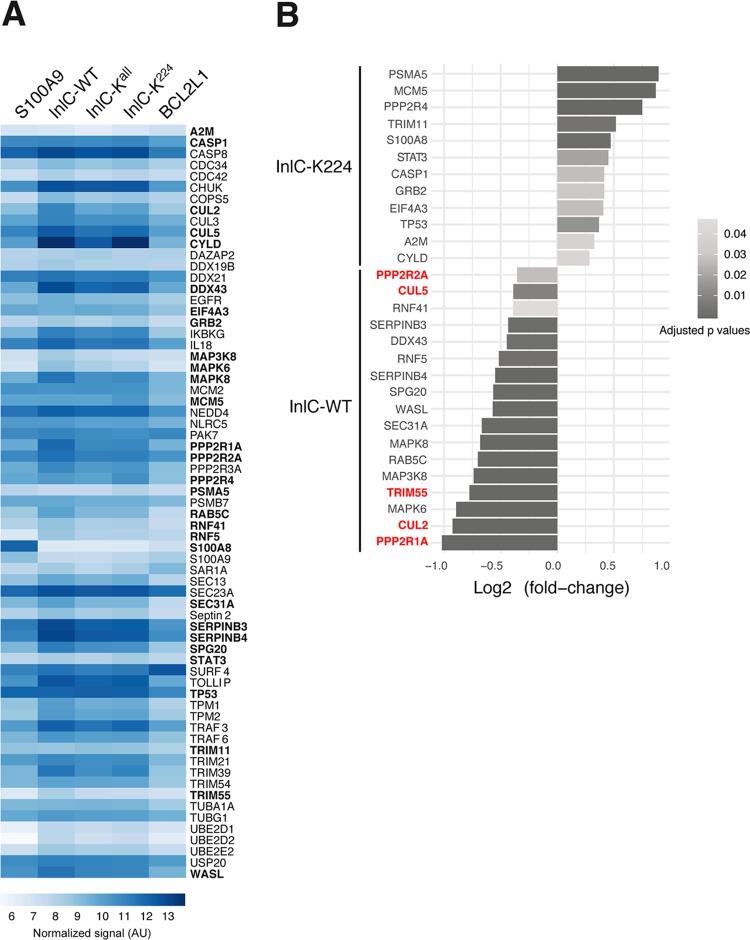
Ubiquitinated InlC interacts with S100A9 partners. (A) Heat map representing interactions of cellular proteins with native InlC, InlC-K^all^, or InlC-K^224^. A total of 67 selected human ORFs were fused to the sequence encoding the Nlc1 fragment of the nanoluciferase in pSNL-N1. The ORFs encoding native InlC, InlC-K^all^, or InlC-K^224^ were fused to the sequence encoding the complementary Nlc2 fragment of the nanoluciferase in pSNL-C2. HEK-293T cells were cotransfected with pSNL-N1 and pSNL-C2, and interactions between each cellular protein and InlC, InlC-K^all^, or InlC-K^224^ were assessed by measuring luciferase activity at 24 h posttransfection. BCL2-L1 was used as a negative control, and S100A9 was used as a positive control. Light blue-to-dark blue colors indicate weak-to-strong interactions. Values of the normalized luciferase signals correspond to the means of log-transformed data. The 29 putative interactors of InlC or InlC-K^224^ are shown in boldface type. The data shown are representative of independent data from three experiments. AU, arbitrary units. (B) Bar plot of the log_2_-fold changes for the 29 proteins interacting preferentially with InlC or InlC-K^224^. Negative values indicate a stronger interaction of the proteins with InlC, and positive values indicate a stronger interaction of the proteins with InlC-K^224^. Five direct partners of S100A9 are shown in red. The gray scale indicates adjusted *P* values. The data shown are representative of independent data from three experiments.

To further assess the functional link between ubiquitinated InlC and S100A9, a list of potential common partners of InlC and S100A9 was established based on the two known interactors of InlC (16, 21), putative interactors of InlC found in a two-hybrid screen (Table S2), and S100A9 interactors found in the BioGRID database (Table S3). A total of 67 cDNAs encoding 67 human factors from the human ORFeome collection (Center for Cancer Systems Biology [CCSB], Dana-Farber Institute, Boston, MA, USA) as clones in the pDONR223 vector were selected. The human open reading frames (ORFs) were transferred by Gateway recombination in fusion with the sequence encoding the Nlc1 fragment of the nanoluciferase into the destination vector pSNL-N1. The ORFs encoding native InlC, InlC-K^all^, or InlC-K^224^ were fused to the sequence encoding the complementary Nlc2 fragment of the nanoluciferase into the vector pSNL-C2. HEK-293T cells were cotransfected with pSNL-N1 and pSNL-C2, and interactions between each partner and the InlC, InlC-K^all^, or InlC-K^224^ protein were assessed by measuring luciferase activity at 24 h posttransfection (Fig. 6A). The human protein BCL2-L1 was used as a negative control. CHUK/IKK-α and Tuba, the two characterized InlC interactors, were used as positive controls. We focused our analysis on the difference in interactions between native InlC and InlC-K^224^. For each potential partner, the difference in the luciferase signals between the pair partner/InlC and the pair partner/InlC-K^224^ was calculated. In total, 29 binary interactions of the 67 pairs were found to be statistically different between InlC and InlC-K^224^, among which 17 pairs show a reduction of interaction when lysine residue 224 was mutated (Fig. 6B). Thus, these proteins could interact better with ubiquitinated InlC than with InlC-K^224^. On the other hand, we identified 12 pairs with an increased interaction when InlC was not ubiquitinated (Fig. 6B). Among the 29 interactors, 5 proteins are known to be direct ligands of S100A9: cullin-2 (Cul2) and cullin-5, two members of the cullin-RING E3 ubiquitin ligase (CRL) complexes; the PPP2R1A and PPP2R2A subunits of serine/threonine phosphatases; and TRIM55, a protein with a tripartite motif. These results indicate that these proteins could be involved in the interaction between InlC and the mammalian host alarmin S100A9.

10.1128/mBio.02778-19.4 TABLE S2 Putative and known InlC interactors identified by yeast two-hybrid screening. Download Table S2, DOC file, 0.03 MB. Copyright © 2019 Gouin et al. 2019 Gouin et al. This content is distributed under the terms of the Creative Commons Attribution 4.0 International license.

10.1128/mBio.02778-19.5 TABLE S3 Putative and known InlC and/or S100A9 interactors. Download Table S3, DOC file, 0.04 MB. Copyright © 2019 Gouin et al. 2019 Gouin et al. This content is distributed under the terms of the Creative Commons Attribution 4.0 International license.

## DISCUSSION

During infection, pathogenic bacteria are constantly struggling with their host for survival and must overcome challenges such as nutritional, innate, and adaptative immunity. To counteract these defenses, intracellular pathogens have developed sophisticated virulence strategies. Some virulence factors target host immune signaling pathways or interfere with the inflammatory process (31). Other bacterial effectors can exploit posttranslational modification machineries of the cell for their own benefit to promote virulence (32–34). However, bacterial effectors can also be modified posttranslationally to be degraded or relocated to contribute to miscellaneous functions (29, 32, 35, 36). Here, we report that the *Listeria* virulence protein InlC is ubiquitinated at late time points during cell infection. This modification is a monoubiquitination on the lysine residue K^224^. Ubiquitination of bacterial virulence factors can lead to their proteasomal degradation, which either promotes or limits infection. For instance, the differential ubiquitination and subsequent degradation of the Salmonella enterica guanine exchange factor SopE and the GTPase-activating protein SptP allow fine-tuning of their respective activities, which is important for bacterial invasion into nonphagocytic cells (37). In contrast, ubiquitination of the Pseudomonas aeruginosa effector ExoT by the host E3 ligase Cbl-b and its proteasomal degradation limit bacterial dissemination (38). Besides its role in promoting protein degradation, ubiquitination has also been shown to affect the function of bacterial effectors. For instance, the *Salmonella* phosphoinositide phosphatase SopB is localized at the plasma membrane at early stages of infection to mediate bacterial entry into cells. At later stages of infection, SopB relocates to the *Salmonella*-containing vacuole upon monoubiquitination on multiple lysine residues by the host E3 ligase TRAF6, promoting intracellular bacterial replication (29, 39). We demonstrate that ubiquitination of InlC does not lead to its degradation but promotes its interaction with the alarmin S100A9, a potent modulator of inflammatory processes (40). This interaction stabilizes S100A9 within the cell, while the InlC-K^224^ mutated protein interacts weakly with S100A9, which is rapidly degraded. InlC ubiquitination and binding to S100A9 occur at late stages of infection compared to InlC binding to the kinase IKK-α, an interaction that dampens the NF-κB-dependent inflammatory process at early stages of infection (16). S100A9 is an important DAMP with intra- and extracellular effects, e.g., antimicrobial and chemotactic activities. During infection, bacterial effectors can induce S100A8/S100A9 expression, which is released by activated phagocytes. Heterodimerization and translocation of S100A9 to the membrane are facilitated by a protein kinase C-dependent rise in the Ca^2+^ level. Protein kinase C activation is also required for S100A9 secretion via the microtubule network (41, 42). In addition, S100A9 can be modified by p38 mitogen-activated protein kinase-dependent threonine phosphorylation, which abrogates tubulin polymerization (43). Thus, intracellularly, the interaction of calprotectin, the S100A8/S100A9 heterodimer, with microtubules regulated by p38 and Ca^2+^ signaling pathways is critical for cytoskeleton reorganization and phagocyte migration (43). Extracellularly, S100A8/S100A9 binds to and activates Toll-like receptor 4 (TLR4) on numerous cell types of the innate immune system. These activated cells then release inflammatory mediators, thereby amplifying inflammatory reactions (55). Calprotectin also mediates the production of ROS by neutrophils (44, 45). We found that infection with the strain producing InlC-K^224^, which leads to rapid S100A9 degradation, triggers weaker ROS production by neutrophils than the WT strain. Furthermore, this phenotype is abolished in S100A9^−/−^ mice, strongly suggesting that the interaction of S100A9 with native InlC could promote ROS production upon infection.

Although the enzymes catalyzing InlC ubiquitination are currently unknown, we identified cullin-2 and cullin-5 as putative partners of InlC by an interactomic screen. Cullin-2 and cullin-5 function as scaffold proteins of cullin-RING E3 ubiquitin ligase (CRL) complexes that mediate ubiquitination of target proteins. In CRL2 complexes, cullin-2 assembles with the RING protein Rbx1, the elongin B and C adapter proteins, and various substrate recognition subunits (SRSs) such as the von Hippel-Lindau (VHL) tumor suppressor protein (46). CRL2 ligases are involved in multiple biological processes, such as development and tumorigenesis, and are subverted by several viruses. In CRL5 complexes, cullin-5 interacts with the RING protein Rbx2, elongins B and C, and suppressor of cytokine signaling (SOCS) box-containing proteins (47). As for CRL2, CRL5 ligases regulate multiple signaling pathways, such as cell proliferation and angiogenesis, and can be hijacked by viruses. CRL2- and CRL5-interacting proteins have domains called the VHL box and the SOCS box, respectively. The VHL box is composed of a cullin-2 box with the consensus sequence ΦPXXΦXXXΦ (where Φ can be any hydrophobic amino acid) and a BC box that binds elongins B and C and has the consensus sequence (S/T/P)LXXX(C/S/A)XXXΦ (48). The SOCS box is composed of a BC box and a cullin-5 box corresponding to the canonical sequence ΦXXLPΦPXXΦXX(Y/F)(L/I) (48). Interestingly, two domains of the InlC sequence have similarity with the consensus sequences of the BC and cullin boxes (see Fig. S2A in the supplemental material). Similarly to viral proteins containing VHL or SOCS boxes, such as HIV-1 Vif (49) and the adenoviral protein E4orf6 (50), InlC could thus form a complex with cullin-2 and/or cullin-5 during infection. Furthermore, by affinity chromatography, S100A9 was found to interact with cullin-2 and cullin-5 (51). Interestingly, in cells infected by L. monocytogenes expressing InlC lacking the last 33 amino acids, which carry the putative Cul2/Cul5 boxes, InlC is not ubiquitinated (Fig. S2B). InlC could thus be ubiquitinated by CRL complexes, thereby stabilizing S100A9.

10.1128/mBio.02778-19.2 FIG S2 InlC could be ubiquitinated by CRL complexes. (A) InlC contains putative BC and cullin boxes. The cullin-RING E3 ubiquitin ligase complexes CRL2 and CRL5 mediate ubiquitination of target proteins containing the VHL box and the SOCS box, respectively. The VHL box is composed of a BC box and a cullin-2 box. The SOCS box is composed of a BC box and a cullin-5 box. Gray boxes highlight putative BC and cullin boxes of 6 human proteins (HsVHL, von Hippel-Lindau disease tumor suppressor; HsLRR-1, leucine-rich repeat protein 1; HsFEM1B, protein fem-1 homolog B; HsZYG11B, protein zyg-11 homolog B; HsZYG11BL, protein zer-1 homolog; HsPRAME, melanoma antigen preferentially expressed in tumors) aligned with L. monocytogenes EGD InlC. Conserved residues from the consensus sequences are shown in red. Φ indicates any hydrophobic amino acids. (B) Expression of InlC-ΔC-ter in infected cells. HeLa cells were infected for 24 h with L. monocytogenes EGD Δ*inlC* expressing the first 264 amino acids of InlC lacking the putative cullin boxes (InlC-ΔC-ter), EGD Δ*inlC* expressing InlC (InlC), wild-type (WT) EGD, or the EGD *inlC* mutant (Δ*inlC*). Total cell lysates were immunoblotted with anti-InlC antibodies. The arrows highlight the main InlC unmodified form and the InlC^H^ ubiquitinated form. Expectedly, the InlC-ΔC-ter strain expresses a slightly smaller form of unmodified InlC than full-length InlC. In contrast to InlC, InlC-ΔC-ter is not ubiquitinated in infected cells, as InlC^H^ is not detected, suggesting that ubiquitination of InlC may require interactions with cullins. Download FIG S2, JPG file, 0.6 MB. Copyright © 2019 Gouin et al. 2019 Gouin et al. This content is distributed under the terms of the Creative Commons Attribution 4.0 International license.

In conclusion, we have shown that InlC is monoubiquitinated on lysine 224 during infection. Host ubiquitination of InlC stabilizes S100A9, triggers ROS production by neutrophils, and restricts infection. Our data reinforce the importance of posttranslational modifications in the subtle regulation of host-pathogen interactions during the infectious process.

## MATERIALS AND METHODS

### Cell culture and infection.

HeLa (human epithelial cervix carcinoma; ATCC CCL 2) cells and human kidney HEK-293T cells (Invitrogen) were cultured according to American Type Culture Collection guidelines. Cells were generally infected with exponentially growing *Listeria* strains such that the multiplicity of infection (MOI) was 50 bacteria per cell (MOI of 50). After 1 h of infection, cells were washed in phosphate-buffered saline (PBS) and treated with 20 μg/ml of gentamicin to prevent the growth of extracellular bacteria.

### Bacterial strains and growth conditions.

References and information on strains can be found in Table S4 in the supplemental material. *Listeria* strains were grown in brain heart infusion (BHI) broth (BD) at 37°C at 200 rpm.

10.1128/mBio.02778-19.6 TABLE S4 Strains used in this study. Download Table S4, DOC file, 0.04 MB. Copyright © 2019 Gouin et al. 2019 Gouin et al. This content is distributed under the terms of the Creative Commons Attribution 4.0 International license.

### Plasmids.

References and information on plasmids can be found in Table S5. For mammalian transfection, the pUC57-FLAG_2_-InlC, pUC57-FLAG_2_-InlC-K^all^, and pUC57-FLAG_2_-InlC-K^224^ plasmids encoding N-terminal FLAG_2_-tagged InlC sequences were optimized for expression in mammalian cells and mutated on lysine residues (GeneCust). InlC-K^all^ refers to the construct where all lysines from K^57^ to K^296^ were replaced by arginines, and InlC-K^224^ refers to lysine 224 replaced by arginine. These constructs were subcloned into pcDNA3 (Invitrogen) at BamHI/XhoI sites. The yeast two-hybrid screen was performed by Hybrigenics using plasmids derived from pBTM116 expressing *inlC* and pGADGH expressing a human placenta gene bank (16).

10.1128/mBio.02778-19.7 TABLE S5 Plasmids used in this study. Download Table S5, DOC file, 0.1 MB. Copyright © 2019 Gouin et al. 2019 Gouin et al. This content is distributed under the terms of the Creative Commons Attribution 4.0 International license.

The split-nanoluciferase complementation assay (NPCA) to perform the interactome screen is based on the ability of interacting protein pairs expressed in fusion with the Nlc1 and Nlc2 complementary fragments of the NanoLuc luciferase to reconstitute an active enzyme. These fragments were positioned at either the N or C terminus of the selected protein. The ORFs encoding potential factors selected for the screen, including S100A8 and S100A9 factors, were obtained from the human ORFeome collection (CCSB, Dana-Farber Institute, Boston, MA, USA) as entry clones in the pDONR223 vector, and they were fused to the Nlc1 fragment of the luciferase. The InlC plasmids had to be fused to the complementary Nlc2 fragment positioned at either the N or C terminus of the InlC constructs to subsequently select the best pairs and obtain the strongest signal in the luciferase assay. The InlC, InlC-K^all^, and InlC-K^224^ ORFs were amplified by PCR from the respective pUC57 constructs (see above) with oligonucleotides harboring Gateway recombination *att*B1.2 and *att*B2.1 sites listed in Table S6 and cloned by *in vitro* recombination into pDONR207 (BP cloning reaction; Invitrogen). Next, the resulting entry clones were transferred by Gateway recombination into the destination vector pSNL-N2 or pSNL-C2 expressing the Nlc2 fragment of NanoLuc luciferase in fusion at either their N or C termini.

10.1128/mBio.02778-19.8 TABLE S6 Primers used in this study. Download Table S6, DOCX file, 0.02 MB. Copyright © 2019 Gouin et al. 2019 Gouin et al. This content is distributed under the terms of the Creative Commons Attribution 4.0 International license.

### Construction of strains expressing mutated InlC.

All PCRs were carried out using the *Pfu* Turbo DNA polymerase (Agilent) according to the manufacturer’s recommendations. The *inlC* gene and its own promoter were PCR amplified from the L. monocytogenes EGD chromosome using primers 1 and 2 and primers 3 and 4, respectively (Table S6), and cloned into the pCR-Blunt vector (Invitrogen) according to the manufacturer’s recommendations, yielding pBlunt-InlC and pBlunt-InlC-K^272,295^, respectively (Table S5). These plasmids were then used as the templates for PCR site-directed mutagenesis using overlapping primers, both containing the desired mutation (Table S6). Extension of the primers generates a mutated pCR-Blunt plasmid containing staggered nicks. PCR products were treated with DpnI to remove the parental DNA template and then transformed into XL1-Blue electrocompetent cells. Transformants were selected on kanamycin, and plasmids containing the expected punctual mutations were verified by sequencing using the M13Fw and -Rev primers. The mutated *inlC* genes encoding InlC without any lysine residues (InlC-K^all^) or without any lysine residues in the LRR region (InlC-K^LRR^) were synthesized and cloned into XmaI-SalI-digested plasmid pUC57 (GeneCust, Luxembourg). The SalI-XmaI restriction fragments, from pBlunt-based or pUC57-based plasmids, composed of either native *inlC* (pBlunt-InlC) or mutated *inlC* were subcloned into the SalI-XmaI-digested pAD_2_-P*inlC*-GFP plasmid (Table S5) (52). All pAD-based plasmids were verified by sequencing using primers pPL2-Rev and pPL2-Fw and transformed into L. monocytogenes EGD Δ*inlC* (BUG2117) by electroporation (Table S4). Integration into the chromosome was verified by PCR amplification using primers NC16 and PL95 (Table S6). For multiple-site mutagenesis, newly synthesized pCR-Blunt-based plasmids containing the *inlC* gene with a punctual mutation(s) were used as the templates for subsequent PCR site-directed mutagenesis as described above (Table S5). For the generation of the L. monocytogenes EGD strain producing InlC lacking the C terminus, the first 792 nucleotides of the *inlC* gene and its own promoter were amplified by PCR from the L. monocytogenes EGD chromosome and cloned into the SmaI/SalI-digested pAD_2_-P*inlC*-GFP plasmid (Table S5). The resulting plasmid, pAD-InlC-T5, was introduced into L. monocytogenes EGD Δ*inlC* (BUG2117) by electroporation (Table S4). The chromosomal insertion was verified by PCR using primers NC16 and PL95 (Table S6).

### Immunoblotting.

Cells were lysed in Laemmli buffer, and proteins eluted from immunoprecipitation assay mixtures were separated on SDS-polyacrylamide gels. Proteins were transferred to polyvinylidene fluoride membranes and incubated with primary antibodies. Membranes were then incubated with anti-rabbit or anti-mouse horseradish peroxidase (HRP)-conjugated antibodies (AbCys). The blots were revealed using the ECL kit or ECL-2 kit (Thermo Fisher Scientific) or the Clarity kit (Bio-Rad).

### Transfection.

HeLa cells were seeded into T75 flasks in complete medium at a density of 2.5 × 10^6^ cells. Cells were transfected the day after with 15 μg of expression plasmids using Lipofectamine LTX reagents according to the manufacturer’s protocol (Invitrogen). After 24 or 48 h, the transfected cells were either directly lysed and used for immunoprecipitation assays or infected as described above, and 24 h later, the transfected/infected cells were lysed and processed for immunoprecipitation.

### Antibodies.

The primary antibodies used in this study were affinity-purified rabbit polyclonal anti-InlC (R134) (16), mouse antiubiquitin (catalog number 3936; Cell Signaling Technology), rabbit anti-SUMO1 (catalog number 4930; Cell Signaling Technology), mouse anti-ISG15 (F-9, catalog number sc166755; Santa Cruz Biotechnology), mouse anti-HA (6E2, catalog number 2367; Cell Signaling Technology), rabbit anti-HA (C29F4; Cell Signaling Technology), mouse antiactin (AC-15; Sigma-Aldrich), rabbit anti-S100A9 (catalog number pab0423-P; Covalab), and mouse anti-NF-κB-p65 (F6; Santa Cruz).

### ELISA.

HeLa cells were cultured in 24-well plates according to American Type Culture Collection guidelines. Cells were transfected with pcDNA3.1+/N-HA-S100A9 at 500 ng/well (Table S5). After 48 h, cells were infected with exponentially growing *Listeria* strains such that the multiplicity of infection was 60 bacteria per cell. Plates were centrifuged for 5 min at 1,100 rpm at 18°C and incubated at 37°C for 90 min. Cells were washed 3 times in PBS, treated with 20 μg/ml of gentamicin, and stimulated with 50 ng/ml of human TNF-α (R&D Systems). After 24 h, S100A9 secreted by infected cells was quantitated by an enzyme-linked immunosorbent assay (ELISA) according to the manufacturer’s recommendations (DuoSet ELISA S100A9; R&D Systems).

### Split-nanoluc luciferase complementation assay.

The luciferase protein complementation assay was performed in a 96-well-plate format (Greiner, Kremsmünster, Austria) using HEK-293T cells (40,000 cells/well plated in 100 μl of Dulbecco’s modified Eagle’s medium [DMEM] supplemented with 10% fetal bovine serum and antibiotics). After 24 h, cells were transfected using a polyethylenimine (PEI) method with 200 ng total DNA mix containing plasmid pairs respectively encoding the human proteins fused at the N or C terminus to amino acids 1 to 65 of the nanoluciferase and with a plasmid bearing the N or C terminus fused to the complementary moieties of luciferase and encoding either S100A9, InlC proteins, or an irrelevant protein (BCL2-L1) as a negative control. At 24 h posttransfection, the medium of the cells was harvested, and the cells were incubated with 50 μl of Nano-Glo luciferase reagent (Promega). Luminescence monitoring was performed on a Centro XS3 LB 960 microplate luminometer (Berthold Technologies) using an integration time of 2 s. After cotransfection of the different constructs, the highest efficiency of interaction revealed was obtained when these moieties were placed at the C terminus of S100A9 and InlC, giving the combination C1-S100A9/C2-InlC. For each interaction, *P* values were calculated from the means of quadruplicates, using the Holm-Sidak *t* test.

### Immunoprecipitation.

Infected or transfected cells were rinsed three times in cold PBS. For immunoprecipitation of infected cells, cells were lysed for 2 h in modified radioimmunoprecipitation assay (RIPA) buffer (50 mM Tris-HCl [pH 7.6], 1% NP-40, 0.5% sodium deoxycholate, 150 mM NaCl, 0.1% sodium dodecyl sulfate, 1 mM EDTA, 20 mM *N*-ethylmaleimide, and a complete protease inhibitor cocktail [Roche]). Lysates were centrifuged for 15 min at 4°C at 16,000 × *g*, and supernatants were incubated for 15 min with protein A-Sepharose beads (GE Healthcare) to eliminate unspecific binding of proteins to beads. After the removal of the beads, cleared lysates were then incubated overnight at 4°C with anti-InlC antibodies. The immunocomplexes were then captured by incubating samples for 3 h with protein A-Sepharose beads. Beads were finally collected and washed four times in the corresponding lysis buffer, and captured proteins were eluted using Laemmli buffer. For immunoprecipitation of FLAG-tagged InlC, cells were lysed for 2 h in FLAG lysis buffer (20 mM Tris-HCl [pH 8.0], 1% Triton, 150 mM NaCl, and a complete protease inhibitor cocktail [Roche]). Lysates were centrifuged, and 1.5 mg of the total extract was incubated overnight at 4°C with 15 μl of washed anti-FLAG magnetic beads (Sigma-Aldrich). The immunocomplexes were washed four times with buffer containing 0.25% Triton by using a magnetic rack, and proteins were eluted twice using 20 μl of 3× FLAG peptide (100 μg/ml of 3× FLAG peptide [Sigma-Aldrich] in a solution containing 50 mM Tris-HCl [pH 7.4] and 150 mM NaCl).

### Murine infection experiments.

L. monocytogenes strains were thawed from glycerol stocks stored at −80°C, washed, and diluted in PBS before injection. LD_50_ experiments were carried out by injecting 200-μl serial dilutions of the bacterial suspension intravenously in the tail vein of 8-week-old female BALB/c mice (Charles River). LD_50_ values were determined by the probit method after infection of groups of 5 mice. Kaplan-Meier curves were generated by measuring mouse daily survival over 10 days. For peritoneal infections, a sublethal dose (10^7^ bacteria) was injected into the peritoneal cavity of 8-week-old female C57BL/6 mice (Charles River) or S100A9^−/−^ mice, provided by Thomas Vogl (53). The inoculum was confirmed by plating serial dilutions of the bacterial suspension on BHI agar plates. For the determination of bacterial loads, peritoneal lavage fluids were recovered at 24, 48, and 72 h postinfection. Serial dilutions of organ homogenates were plated on BHI agar plates, and CFU were counted after growth at 37°C for 48 h. All experiments were performed in accordance with the Institut Pasteur’s guidelines for laboratory animal welfare. For flow cytometry, peritoneal lavage fluids were washed in staining buffer (PBS with 0.5% fetal calf serum [FCS] and 2 mM EDTA), counted, and distributed in 96-well plates for staining. Unspecific binding was blocked by incubation with anti-mouse CD16/CD32 (BD Biosciences) for 10 min before the addition of surface-labeling antibodies for another 40 min, in staining buffer at 4°C. Cells were washed twice in PBS before viability dye (eFluor780; eBioscience) labeling for 5 min at 4°C. Cells were washed twice in staining buffer and fixed for 5 min using a commercial fixation buffer (BioLegend). For intracellular cytokine staining, cells were permeabilized and washed with buffers from commercial kits (Inside stain kit; Miltenyi Biotec). For interferon gamma (IFN-γ) detection, samples were incubated for 4 h at 37°C with brefeldin A (eBioscience) before staining. ROS detection was performed according to the manufacturer’s instructions (Total ROS assay kit 520 nm; Invitrogen). The following antibodies (clones) were used: NK1.1 (PK136), CD3 (145-2C11), CD11b (M1/70), CD69 (H1.2F3), CD25 (PC61), Ly6C (HK1.4), Ly6G (1A8-Ly6g), CD11c (HL3), CD64 (X54-5/7.1), F4/80 (T45-2342), NOS2 (CXNFT), and IFN-γ (XMG1.2) (purchased from BioLegend, eBioscience, and BD Biosciences). Sample acquisition was performed on a MACSQuant cytometer (Miltenyi Biotec), and analysis was done using FlowJo software (TreeStar).

### Ethics statement.

This study was carried out in strict accordance with French national and European laws and conformed to the Council Directive on the approximation of laws, regulations, and administrative provisions of the Member States regarding the protection of animals used for experimental and other scientific purposes (86/609/EEC). Experiments that relied on laboratory animals were performed in strict accordance with the Institut Pasteur’s regulations for animal care and use protocol, which was approved by the Animal Experiment Committee of the Institut Pasteur (approval number 03-49).

### Statistical analysis.

To compare the interactions between InlC, InlC-K^224^, and their putative partners, we performed statistical analysis with the limma package (v3.30.13) and voom transformation (54). The data were normalized using trimmed mean of M values (TMM) normalization. By shrinking the variance estimates toward a common value, the linear model implemented in the limma package provides a robust comparison for each protein between the two conditions InlC and InlC-K^224^. The resulting *P* values were adjusted according to the Benjamini-Yekutieli procedure (56).
