# Supplementary figures and images for: Ubiquitination of Listeria Virulence Factor InlC Contributes to the Host Response to Infection
Source: mBio. 2019 Dec 17;10(6):e02778-19. doi: 10.1128/mBio.02778-19 (PMC6918085; doi:10.1128/mBio.02778-19)

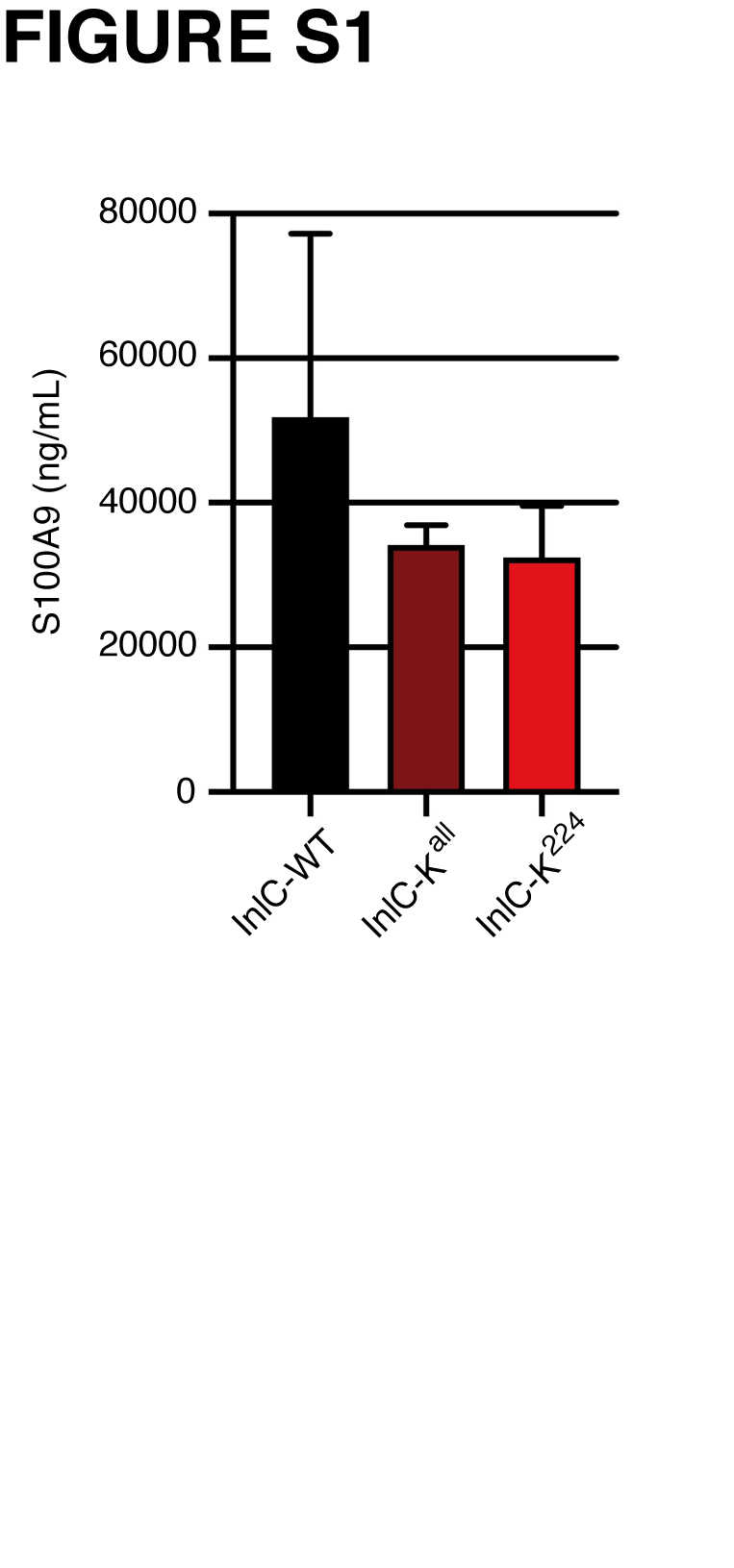

Supplement: FIG S1 [file mBio.02778-19-sf001.tif]

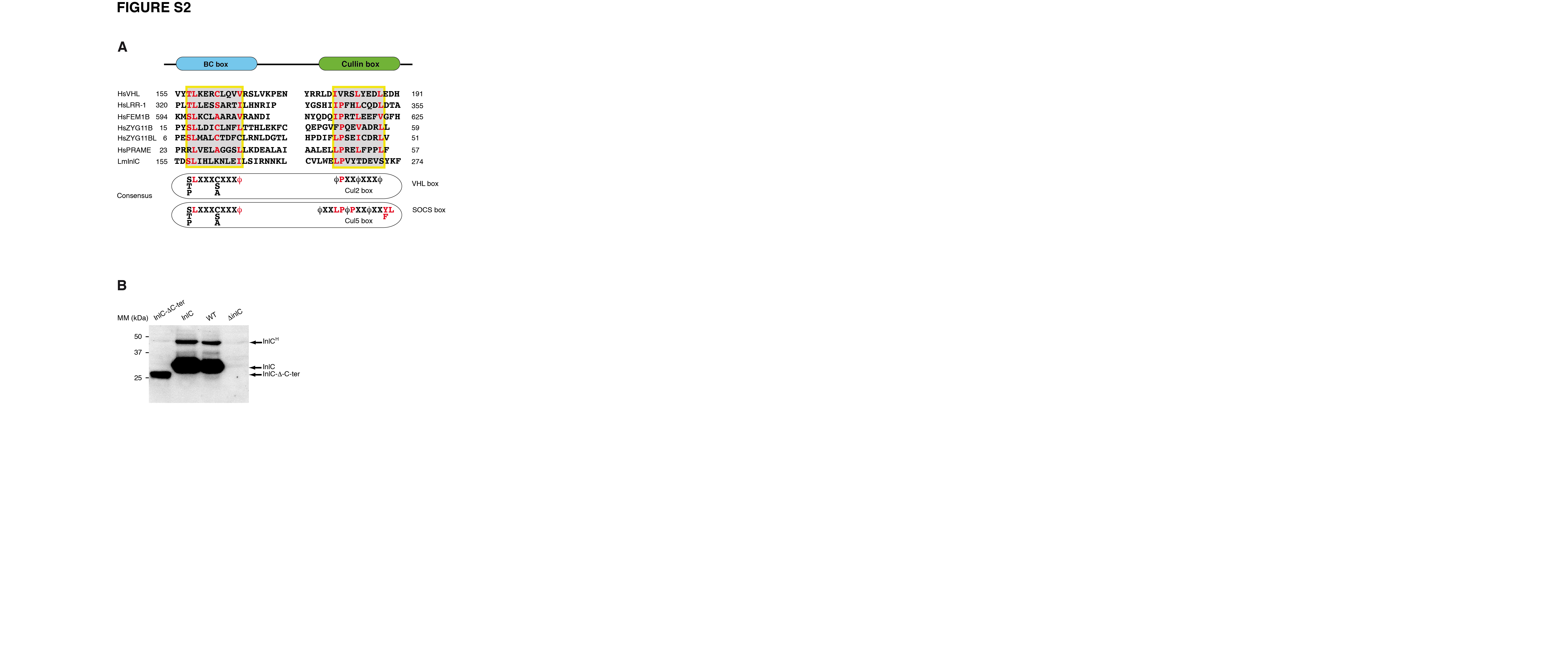

Supplement: FIG S2 [file mBio.02778-19-sf002.jpg]
