# Supplementary material for: Ubiquitination of Listeria Virulence Factor InlC Contributes to the Host Response to Infection
Source: mBio. 2019 Dec 17;10(6):e02778-19. doi: 10.1128/mBio.02778-19 (PMC6918085; doi:10.1128/mBio.02778-19)
Supplement: TABLE S2 [file mBio.02778-19-st002.doc]

**Table S2. Putative and known InlC interactors identified by yeast two-hybrid screen**

| **Gene** | **Protein** | **Source/Reference** |
| --- | --- | --- |
| A2M  CHUK  CTNNAL1  DAZAP2  N4BP3  PPP2R3A  RNF5  SPG20  TPR  TRIM39  TUBA | Alpha-2 macroglobulin  Component of inhibitor of nuclear factor kappa-B kinase complex (IKK-alpha)  Alpha-catulin  Deleted in azoospermia-associated protein 2  NEDD4-binding protein 3  Protein phosphatase 2 regulatory subunit B  E3 ubiquitin-protein ligase ring finger protein 5  Spartin  Translocated promoter region protein (Megator)  E3 ubiquitin-protein ligase tripartite motif containing 39  Tubulin alpha | This study  Gouin *et al.* 2010  This study  This study  This study  This study  This study  This study  This study  This study  Rajabian *et al.* 2009 |
