# Supplementary material for: Ubiquitination of Listeria Virulence Factor InlC Contributes to the Host Response to Infection
Source: mBio. 2019 Dec 17;10(6):e02778-19. doi: 10.1128/mBio.02778-19 (PMC6918085; doi:10.1128/mBio.02778-19)
Supplement: TABLE S3 [file mBio.02778-19-st003.doc]

**Table S3. Putative and known InlC and/or S100A9 interactors**

| **Gene** | **Protein** | **Interactor (degree)*** |
| --- | --- | --- |
| A2M  CASP1  CASP8  CDC34  CDC42  CHUK  COPS5  CUL2  CUL3  CUL5  CYLD  DAZAP2  DDX19B  DDX21  DDX43  EGFR  EIF4A3  GRB2  IKBKG  IL18  MAP3K8  MAPK6  MAPK8  MCM2  MCM5  NEDD4  NLRC5  PAK7  PPP2R1A  PPP2R2A  PPP2R3A  PPP2R4  PSMA5  PSMB7  RAB5C  RNF41  RNF5  S100A8  S100A9  SAR1A  SEC13  SEC23A  SEC31A  SEPT2  SERPINB3  SERPINB4  SPG20  STAT3  SURF4  TOLLIP  TP53  TPM1  TPM2  TRAF3  TRAF6  TRIM11  TRIM21  TRIM39  TRIM54  TRIM55  TUBA1A  TUBG1  UBE2D1  UBE2D2  UBE2E2  USP20  WASL | Alpha-2 macroglobulin  Caspase 1 apoptosis-related cysteine peptidase  Caspase 8 apoptosis-related cysteine peptidase  Cell division cycle 34  Cell division cycle 42  Component of inhibitor of nuclear factor kappa-B kinase complex  COP9 signalosome subunit 5  Cullin 2  Cullin 3  Cullin 5  Cylindromatosis  Deleted in azoospermia-associated protein 2  DEAD box polypeptide 19B  DEAD box helicase 21  DEAD box polypeptide 43  Epidermal growth factor receptor  Eukaryotic translation initiation factor 4A3  Growth factor receptor-bound protein 2  Inhibitor of kappa light polypeptide gene enhancer in B-cells kinase gamma  Interleukin 18  Mitogen-activated protein kinase kinase kinase 8  Mitogen-activated protein kinase 6  Mitogen-activated protein kinase 8  Minichromosome maintenance complex component 2  Minichromosome maintenance complex component 5  Neural precursor cell expressed developmentally down-regulated 4  NLR family CARD domain containing 5  p21 protein (CDC42/RAC)-activated kinase 7  Protein phosphatase 2 regulatory subunit A alpha  Protein phosphatase 2 regulatory subunit B alpha  Protein phosphatase 2 regulatory subunit B" alpha  Protein phosphatase 2 regulatory subunit 4  Proteasome subunit alpha type 5  Proteasome subunit beta type 7  RAS oncogene family member RAB5C  E3 ubiquitin-protein ligase ring finger protein 41  E3 ubiquitin-protein ligase ring finger protein 5  S100 calcium binding protein A8  S100 calcium binding protein A9  Secretion-associated RAS-related GTPase 1A  S. cerevisiae SEC13 homolog  S. cerevisiae SEC23 homolog A  S. cerevisiae SEC31 homolog A  Septin 2  Serpin peptidase inhibitor clade B member 3  Serpin peptidase inhibitor clade B member 4  Spartin  Signal transducer and activator of transcription 3  Surfeit 4  Toll interacting protein  Tumor protein p53  Tropomyosin 1  Tropomyosin 2  TNF receptor-associated factor 3  E3 ubiquitin-protein ligase TNF receptor-associated factor 6  Tripartite motif containing 11  Tripartite motif containing 21  E3 ubiquitin-protein ligase tripartite motif containing 39  Tripartite motif containing 54  Tripartite motif containing 55  Tubulin alpha 1A  Tubulin gamma 1  Ubiquitin-conjugating enzyme E2D1  Ubiquitin-conjugating enzyme E2D2  Ubiquitin-conjugating enzyme E2E2  Ubiquitin specific peptidase 20  Wiskott-Aldrich syndrome-like | InlC (1)  S100A9 (2)  InlC (2)  S100A9 (1)  S100A9 (1)  InlC (1)  S100A9 (1)  S100A9 (1)  InlC (2)  S100A9 (1)  S100A9 (1)  InlC (1)  S100A9 (1)  S100A9 (1)  S100A9 (2)  S100A9 (1)  S100A9 (1)  S100A9 (1)  InlC (2)  S100A9 (2)  InlC (2)  InlC (2)  S100A9 (2)  S100A9 (1)  S100A9 (1)  InlC (2)  InlC (2)  S100A9 (1)  S100A9 (1)  S100A9 (1)  InlC (1)  InlC (2) S100A9 (2)  S100A9 (1)  S100A9 (1)  S100A9 (2)  InlC (2)  InlC (1)  S100A9 (1)  InlC (1) S100A9 (1)  S100A9 (2)  S100A9 (2)  S100A9 (2)  S100A9 (2)  InlC (2) S100A9 (2)  InlC (1)  InlC (1)  InlC (1)  S100A9 (2)  S100A9 (1)  InlC (2) S100A9 (2)  InlC (2) S100A9 (2)  InlC (1)  InlC (1)  S100A9 (2)  InlC (2) S100A9 (2)  InlC (3) S100A9 (3)  InlC (2) S100A9 (2)  InlC (1)  S100A9 (2)  S100A9 (1)  InlC (1) S100A9 (1)  InlC (2)  InlC (2) S100A9 (2)  InlC (2) S100A9 (2)  InlC (2) S100A9 (2)  InlC (2) S100A9 (2)  InlC (2) |

*InlC first degree interactors were identified by yeast two-hybrid screen or affinity capture-mass spectrometry. InlC second degree interactors and S100A9 interactors were selected in the BioGRID database.
