# Supplementary material for: Ubiquitination of Listeria Virulence Factor InlC Contributes to the Host Response to Infection
Source: mBio. 2019 Dec 17;10(6):e02778-19. doi: 10.1128/mBio.02778-19 (PMC6918085; doi:10.1128/mBio.02778-19)
Supplement: TABLE S4 [file mBio.02778-19-st004.doc]

**Table S4. Strains used in this study**

| **Strain** | **Description** | **Collection #** | **Source or reference** |
| --- | --- | --- | --- |
| EGD | *Listeria monocytogenes* EGD strain | BUG600 | Gouin *et al.,* 2010 |
| ∆*inlC* | EGD∆*inlC* | BUG2117 | Gouin *et al.,* 2010 |
| ∆*inlC-inlC* | EGD∆*inlC* (pAD-InlC) | BUG2615 | Gouin *et al.,* 2010 |
| ∆*inlC*-*inlC*∆C-ter | EGD∆*inlC* (pAD-InlC-T5) | BUG2613 | This study |
| K^57,62^ | EGD∆*inlC* (pAD-InlC-K^57,62^) | BUG2919 | This study |
| K^72^ | EGD∆*inlC* (pAD-InlC-K^72^) | BUG3451 | This study |
| K^173^ | EGD∆*inlC* (pAD-InlC-K^173^) | BUG3084 | This study |
| K^224^ | EGD∆*inlC* (pAD-InlC-K^224^) | BUG2959 | This study |
| K^173,224^ | EGD∆*inlC* (pAD-InlC-K^173,224^) | BUG3086 | This study |
| K^217,236,273,296^ | EGD∆*inlC* (pAD-InlC-K^217,236,273,296^) | BUG2923 | This study |
| K^216,224,236,273,296^ | EGD∆*inlC* (pAD-InlC-K^216,224,236,273,296^) | BUG2924 | This study |
| K^LLR^ | EGD∆*inlC* (pAD-InlC-K^LRR^) | BUG2925 | This study |
| K^all^ | EGD∆*inlC* (pAD-InlC-K^all^) | BUG2926 | This study |
