# Supplementary material for: Ubiquitination of Listeria Virulence Factor InlC Contributes to the Host Response to Infection
Source: mBio. 2019 Dec 17;10(6):e02778-19. doi: 10.1128/mBio.02778-19 (PMC6918085; doi:10.1128/mBio.02778-19)
Supplement: TABLE S5 [file mBio.02778-19-st005.doc]

**Table S5. Plasmids used in this study**

| **Plasmids** | **Characteristics** | **Collection number** | **Reference** |
| --- | --- | --- | --- |
| pCR®-Blunt | Blunt-end PCR cloning vector |  | Invitrogen |
| pAD_2_-P*inlC*-GFP | pPL2 derivative plasmid expressing GFP under the control of P*inlC* | BUG2491 | Balestrino et al, 2010 |
| pBlunt-InlC | Circular PCR template plasmid encoding *inlC* under the control of P*inlC* |  | This study |
| pAD-InlC | Expression of InlC under the control of P*inlC* | BUG2608 | This study |
| pAD-InlC-T5 | Expression of InlC T5 (AA 1-264) lacking the 265-297 C-terminus under the control of P*inlC* | BUG2606 | This study |
| pAD-InlC-K^57,62^ | Expression of InlC (K57R, K62R) under the control of P*inlC* | BUG2910 | This study |
| pAD-InlC-K^72^ | Expression of InlC (K72R) under the control of P*inlC* | BUG2911 | This study |
| pAD-InlC-K^173^ | Expression of InlC (K173R) under the control of P*inlC* | BUG3083 | This study |
| pAD-InlC-K^224^ | Expression of InlC (K224R) under the control of P*inlC* | BUG2960 | This study |
| pAD-InlC-K^173,K224^ | Expression of InlC (K173R, K224R) under the control of P*inlC* | BUG3085 | This study |
| pAD-InlC-K^273,296^ | Expression of InlC (K273, 296R) under the control of P*inlC* | BUG 2946 | This study |
| pAD-InlC-K^217,236,273,296^ | Expression of InlC (K217R, K236R, K273R, K296R) under the control of P*inlC* | BUG2915 | This study |
| pAD-InlC-K^217,224,236,273,296^ | Expression of InlC (K217R, K224R, K236R, K273R, K296R) under the control of P*inlC* | BUG2916 | This study |
| pAD-InlC-K^LRR^ | Expression of InlC with K to R substitutions at positions 100, 116, 120, 133, 162, 173, 175, 185, 206, 207 | BUG2917 | This study |
| pAD-InlC-K^all^ | Expression of InlC with K to R substitutions at positions 57, 62, 72, 100, 116, 120, 132, 162, 173, 175, 185, 206, 207, 217, 224, 236, 273, 296 | BUG2918 | This study |
| pSG5-SUMO1-His-HA | Transfection plasmid expressing mature form of SUMO1 with amino-terminal 6His and HA tags | BUG2863 | Ribet et al, 2010 |
| pcDNA3.1+/N-HA-S100A9 | Transfection plasmid expressing S100A9 (clone ID: OHu25452C) with amino-terminal HA tag | BUG4035 | GenScript |
| pcDNA3-Ubiquitin-HA-K^null^ | Plasmid encoding Ubiquitin HA-tagged lysine-less (Knull) | BUG3023 | Patel et al, 2009 |
| pcDNA3-FLAG_2_-InlC | Plasmid encoding humanized wild type form of InlC with amino-terminal Flag_2_ tag | BUG4039 | This study |
| pcDNA3-FLAG_2_-InlC-K^all^ | Plasmid encoding humanized form of InlC mutated for all lysine residues with amino-terminal Flag_2_ tag | BUG4041 | This study |
| pcDNA3-FLAG_2_-InlC-K^224^ | Plasmid encoding humanized form of InlC mutated on lysine K224 with amino-terminal Flag_2_ tag | BUG4043 | This study |
| pDonor207-InlC-N2 | Gateway pEntry plasmid encoding Nanoluciferase domain fused to humanized wild type form of InlC | BUG4147 | This study |
| pDonor207-inlC-K^all^-N2 | Gateway pEntry plasmid encoding Nanoluciferase domain fused to humanized form of InlC mutated for all lysine residues | BUG4148 | This study |
| pDonor207-inlC-K^224^-N2 | Gateway pEntry plasmid encoding Nanoluciferase domain fused to humanized form of InlC mutated on lysine K224 | BUG4149 | This study |
| pDonor207-InlC-C2 | Gateway pEntry plasmid encoding humanized wild type form of InlC fused to Nanoluciferase domain | BUG4150 | This study |
| pDonor207-InlC-K^all^-C2 | Gateway pEntry plasmid encoding humanized form of InlC mutated for all lysine residues fused to Nanoluciferase domain | BUG4151 | This study |
| pDonor207-InlC-K^224^-C2  pSNL-N2  pSNL-C2  pSNL-N1  pSNL-C1  pUC57-FLAG_2_-InlC-WT  pUC57-FLAG_2_-InlC-K^all^  pUC57-FLAG_2_-InlC-K^224^ | Gateway pEntry plasmid encoding humanized form of InlC mutated on lysine K224 fused to Nanoluciferase domain  Plasmid encoding the nanoluciferase domain 2 in N-terminus  Plasmid encoding the nanoluciferase domain 2 in C-terminus  Plasmid encoding the nanoluciferase domain 1 in N-terminus  Plasmid encoding the nanoluciferase domain 1 in C-terminus  Plasmid encoding humanized *inlC* with amino-terminal Flag_2_ tag  Plasmid encoding humanized *inlC* mutated for all lysine residues with amino-terminal Flag_2_ tag  Plasmid encoding humanized *inlC* mutated on lysine K224 with amino-terminal Flag_2_ tag | BUG4152  BUG4038  BUG4040  BUG4042 | This study  This study  This study  This study  This study  This study  This study  This study |
