# Supplementary material for: Ubiquitination of Listeria Virulence Factor InlC Contributes to the Host Response to Infection
Source: mBio. 2019 Dec 17;10(6):e02778-19. doi: 10.1128/mBio.02778-19 (PMC6918085; doi:10.1128/mBio.02778-19)
Supplement: TABLE S6 [file mBio.02778-19-st006.docx]

**Table S6. Primers used in this study**

| **#** | **Primer name** | **Sequence** | **Use** |
| --- | --- | --- | --- |
| 1 | PinlC-Fw-SmaI | GAGTCACCCGGGATTATTAACGCTTGTTAATTT^1^ | Amplification of *inlC* with its own promoter for cloning into pBlunt |
| 2 | InlC(end)-Rv | GAGTCAGTCGACTTACTAATTCTTGATAGGTTGTGTAAC^1^ |  |
| 3 | PinlC-Fw-SmaI | GAGTCACCCGGGATTATTAACGCTTGTTAATTT^1^ | Generation of the K273R and K296R substitutions |
| 4 | InlC(K273,296R)-Rv | TCAGTCGACTTACTAATTC**C**TGATAGGTTGTGTAACTGTTCCATCAAATATAGCCTCAGTCTCCCCAACGTTTATATATTCGCTAAAC**C**TATAGCTTACTTCATCTGT^1,2^ |  |
| 5 | InlC(K57,62)-Fw | GCGAATGCAGTG**CGT**CAAAATTTAGGGA**GA**CAAAGTGTTACAGACCTTGTAT^2^ | Generation of the K57R and K62R substitutions |
| 6 | InlC(K57,62)-Rv | ACTTTG**TC**TCCCTAAATTTTG**ACG**CACTGCATTCGCTAGGCC^2^ |  |
| 7 | InlC(K72)-Fw | TCACAA**CGT**GAACTATCTGGAGTACAAAATT^2^ | Generation of the K72R substitution |
| 8 | InlC(K72)-Rv | AATTTTGTACTCCAGATAGTTC**ACG**TTGTGATACAAGGTCTGTAACA^2^ |  |
| 9 | InlC(K173)-Fw | TCTATTCGTAATAAT**CG**GTTAAAAAGTATTGTGATGCTT^2^ | Generation of the K173R substitution |
| 10 | InlC(K173)-Rv | CACAATACTTTTTAAC**CG**ATTATTACGAATAGATAAGATT^2^ |  |
| 11 | InlC(K217R)-Fw | GATAGATTTAACTGGTCAGA**G**ATGTGTGAATGAACCAGTA^2^ | Generation of the K217R substitution |
| 12 | InlC(K217R)-Rv | TACTGGTTCATTCACACAT**C**TCTGACCAGTTAAATCTATC^2^ |  |
| 13 | InlC(K224R)-Fw | ATGTGTGAATGAACCAGTA**CGT**TACCAACCAGAATTGTATATAAC^2^ | Generation of the K224R substitution |
| 14 | InlC(K224R)-Rv | GTTATATACAATTCTGGTTGGTA**ACG**TACTGGTTCATTCACACAT^2^ |  |
| 15 | InlC(K236R)-Fw | AATTGTATATAACAAATACTGTCA**G**AGACCCAGATGGAAGATGGA^2^ | Generation of the K236R substitution |
| 16 | InlC(K236R)-Rv | ATCTGGGTCT**C**TGACAGTATTTGTTATATACAATT^2^ |  |
| 17 | M13Fw | TGTAAAACGACGGCCAGT | Sequencing of inserts cloned in pBlunt vector |
| 18 | M13Rev | CAGGAAACAGCTATGACC |  |
| 19 | pPL2-Fw | TTCGACCCGGTCGTCGGTTC | Sequencing of inserts cloned in pAD-based vectors |
| 20 | pPL2-Rev | CTTAGACGTCATTAACCCTCAC |  |
| 21 | NC16 | GTCAAAACATACGCTCTTATC | Verification of pAD integration in the *Listeri*a chromosome |
| 22 | PL95 | ACATAATCAGTCCAAAGTAGATGC |  |
| 23 | Fw-inlCwtCH | GGGGACAACTTTGTACAAAAAAGTTGGCATGGAGAGCATCCAGAGACCTACCCCAATC | Generation of pDonor207-inlCwtNH |
| 24 | Rv-inCwtNH | GGGGACAACTTTGTACAAGAAAGTTGGGtTAGTTCTTAATGGGCTGAGTCACAGTGCC | InlC with *att*B2 site and stop codon |
| 25 | Fw-inlCKallCH | GGGGACAACTTTGTACAAAAAAGTTGGCATGGAGAGCATCCAGAGGCCAACCCCCATC | InlC-K^all^ with *att*B1 site |
| 26 | Rv-inlCKallNH | GGGGACAACTTTGTACAAGAAAGTTGGGtTAATTCCGAATGGGCTGTGTCACTGTGCC | InlC-K^all^ with *att*B2 site and stop codon |
| 27 | Fw-inlCK224CH | GGGGACAACTTTGTACAAAAAAGTTGGCATGGAGTCCATCCAGAGACCAACCCCAAT | InlC-K^224^ with *att*B1 site |
| 28 | Rv-inlCK224NH | GGGGACAACTTTGTACAAGAAAGTTGGGtTAGTTCTTAATGGGCTGTGTCACTGTGCC | InlC-K^224^ with *att*B2 site and stop codon |
| 29 | Rv-inCwtCH | GGGGACAACTTTGTACAAGAAAGTTGGGTTCTTAATGGGCTGAGTCACAGTGCC | InlC with *att*B2 site |
| 30 | Rv-inCKallCH | GGGGACAACTTTGTACAAGAAAGTTGGATTCCGAATGGGCTGTGTCACTGTGCC | InlC-K^all^ with *att*B2 site |
| 31 | Rv-inCK224CH | GGGGACAACTTTGTACAAGAAAGTTGGGTTCTTAATGGGCTGTGTCACTGTGCC | InlC-K^224^ with *att*B2 site |
| 32 | SNL-N2-fw | CGGAGTGACCGGCTGGCGGCTG | Sequencing of pSNL-N2 plasmids |
| 33 | SNL-C2-rev | CGATCTGGCCCATTTGGTC | Sequencing of pSNL-C2 plasmids |
| 34 | SNL-N1-fw | GCTGAAGATCGACATCCATGTC | Sequencing of pSNL-N1 plasmids |
| 35 | SNL-C1-rev | CGGCTGTCTGTCGCCAGTC | Sequencing of pSNL-C1 plasmids |

^1^Underlined nucleotides correspond to the restriction sites *Sal*I and *Xma*I or *att*B1 and *att*B2 Gateway sites.

^2^Bold letters represent the substituted nucleotides changing lysine residue to arginine residue.
